# Light responsive metal–organic frameworks as controllable CO-releasing cell culture substrates†
†Electronic supplementary information (ESI) available: PXRD, XRF and EDX quantification, electron microscopy, N_2_ adsorption isotherms, FTIR and UV-Vis spectroscopic characterization and TGA data. See DOI: 10.1039/c6sc04824b



**DOI:** 10.1039/c6sc04824b

**Published:** 2016-12-21

**Authors:** Stéphane Diring, Arnau Carné-Sánchez, JiCheng Zhang, Shuya Ikemura, Chiwon Kim, Hiroshi Inaba, Susumu Kitagawa, Shuhei Furukawa

**Affiliations:** a Institute for Integrated Cell-Material Sciences (WPI-iCeMS) , Kyoto University , Yoshida, Sakyo-ku , Kyoto 606-8501 , Japan . Email: shuhei.furukawa@icems.kyoto-u.ac.jp ; Email: kitagawa@icems.kyoto-u.ac.jp; b Department of Synthetic Chemistry and Biological Chemistry , Graduate School of Engineering , Kyoto University , Katsura, Nishikyo-ku , Kyoto 615-8510 , Japan

## Abstract

A new carbon monoxide (CO)-releasing material has been developed by embedding a manganese carbonyl complex, MnBr(bpydc)(CO)_3_ (bpydc = 5,5′-dicarboxylate-2,2′-bipyridine) into a highly robust Zr(iv)-based metal–organic framework (MOF). Efficient and controllable CO-release was achieved under exposure to low intensity visible light. Size-controllable nanocrystals of the photoactive MOF were obtained and their CO-releasing properties were correlated with their crystal sizes. The photoactive crystals were processed into cellular substrates with a biocompatible polymer matrix, and the light-induced delivery of CO and its subsequent cellular uptake were monitored using a fluorescent CO-probe. The results discussed here demonstrate a new opportunity to use MOFs as macromolecular scaffolds towards CO-releasing materials and the advantage of MOFs for high CO payloads, which is essential in future therapeutic applications.

## Abstract 2

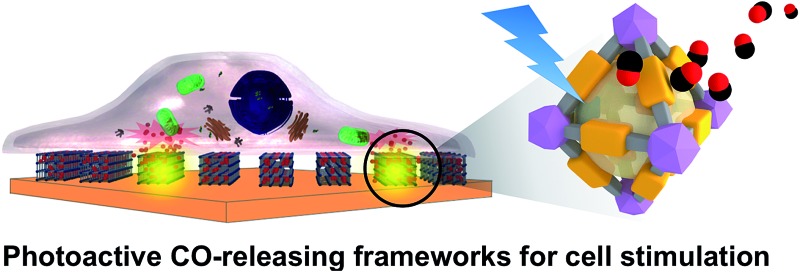
Carbon monoxide-releasing metal–organic frameworks are developed for investigating gas biology at the single cell level.

## Introduction

Carbon monoxide (CO) is not only known for its toxicity, originating from an extraordinary affinity for the haemoglobin heme iron, but it is also an important cell signaling mediator,^1^ classified as a gasotransmitter, akin to nitric oxide and hydrogen sulfide.^2,3^ In addition, CO is known to play a protective role in tissue and organs through its anti-inflammatory, anti-apoptotic, and anti-proliferative properties.^4^ Thus, CO is postulated as an alternative drug to be employed when inflammation plays a damaging role, such as in respiratory and intestinal inflammation.^5^ However, two main challenges have to be addressed before considering CO as a credible therapeutic agent: its systemic toxicity and the issues arising from handling of the gaseous state and the control of the location, dosage and timing of the CO delivery. In this context, stable compounds that are able to store CO in the solid state (or in solution) and liberate it upon external stimuli, are of particular interest. Indeed, light is a non-invasive stimulus that can be manipulated in terms of intensity, wavelength and location. As such, photoactive CO-releasing molecules (photo-CORMs) mostly derived from carbonyl complexes,^6–8^ have been extensively studied and have shown their potential as intracellular CO delivery agents.^9^ However, molecular photoCORMs are difficult to localize due to their fast diffusion after administration,^10^ which may cause toxicity to untargeted healthy tissues, either by the action of the liberated CO or by the release of metal co-ligand fragments after the photoreaction. Therefore, localized CO release in tissues or organs is still challenging.^11^

The hybridization of molecular photoCORMs with macromolecular or inorganic scaffolds is a promising strategy to synthesize photoinduced CO-releasing materials (photoCORMAs) that are easier to localize, which facilitates a tissue-specific therapy.^12^ In addition, the payload of CO in photoCORMAs is higher than in their molecular counterparts, and the extent of leached by-products is limited because the metal carbonyl moiety is immobilized in the scaffold. Thus, to date, photoCORMs have been assembled into dendritic structures,^13^ covalently immobilized onto the surfaces of nanoparticles,^14–16^ protein cages,^17^ or organic polymers,^18^ trapped in polymer fibers,^19^ and mesoporous silica.^20^

In addition to other macromolecular scaffolds, metal–organic frameworks (MOFs) constitute a distinct class of hybrid materials, assembled from metal ions or clusters and organic ligands. Their framework topology, pore size, pore shape and functionality can be modulated by a judicious choice of the molecular components.^21,22^ This modularity in MOF synthesis makes this unique class of materials promising for a wide range of applications in gas storage, molecular separation, catalysis, sensing and drug delivery.^23^ Of particular interest is the possibility to incorporate photodonor ligands as part of the framework scaffold, which helps to maximize the light-harvesting efficiency of photoCORMs by concentrating the photoactive moieties within a restricted space. In addition, the high surface area of MOFs ensures high payloads of photoactive species within the materials. Indeed, we have recently demonstrated the advantage of photoactive MOFs for the controlled delivery of biologically active nitric oxide (NO) molecules at the cellular level.^24,25^ Although there has been only one example of CO-releasing MOF,^26^ in which CO gas is first adsorbed onto open metal sites and then steadily released through ligand exchange reaction with water in physiological media, to the best of our knowledge, highly controllable release of CO from MOFs is yet to be achieved.

Herein, we report how we immobilized a photoactive manganese carbonyl complex within a robust zirconium-based MOF, which demonstrates efficient light-induced CO-release. Using the coordination modulation method,^27,28^ we controlled the MOF crystal sizes with which the CO-donor loading and the photoactive efficiency were correlated. Furthermore, the immobilization of the photoactive MOF particles in a polymer matrix serving as a cell-growth substrate allowed for the observation of intracellular uptake of CO upon visible light irradiation on the substrate.

## Experimental

### Materials and instrumentation

All reagents and reactants were purchased from Wako Pure Chemical Industries and were used without further purification. PXRD measurements were performed using a Rigaku Smartlab (Dtex Ultra detector) operating with a rotating anode Cu Kα X-ray generator (*λ* = 1.54 Å) with a 40 kV beam voltage and 200 mA current. Thermogravimetric analyses (TGA) were carried out in the temperature range from room temperature to 750 °C at a heating rate of 10 °C min^–1^, with a Rigaku Thermo plus EVO2, under nitrogen atmosphere. The sorption isotherms of N_2_ at 77 K were recorded on a BELSORP-max volumetric-adsorption instrument from BEL Japan, Inc. The samples were dried at 50 °C under vacuum conditions for over 12 h before all measurements. Infrared (IR) spectroscopy data were recorded using a Jasco FT/IR-6100 equipped with KBr pellets with 1 cm^–1^ resolution and the accumulation of 64 scans. Samples were observed using a field-emission scanning electron microscope with a JEOL Model JSM-7001F4 system operating at 5 kV and 5 μA current.

### Synthetic procedures

#### Synthesis of UiO-67-bpy

The ligand 2,2′-bipyridine-5,5′-dycarboxylic acid (bpydc) was synthesized according to reported procedures.^29^ DMF solution containing bpydc (20 mM) and ZrCl_4_ (20 mM) and acetic acid (30 or 90 eq.) was prepared and transferred to a Teflon vessel and heated at 120 °C for 24 h. The resulting white microcrystals were recovered by centrifugation and washed with DMF three times. Finally, the crystals were immersed in THF and the solvent was exchanged once every day for three days. Samples were dried under vacuum before loading and sorption experiments.

#### Loading of UiO-67-bpy

Microcrystals of UiO-67-bpy (22 mg, 0.01 mmol) were suspended in a mixture of THF : toluene (1 : 1, 10 ml) containing MnBr(CO)_5_ (25.4 mg, 0.093 mmol) and stirred at room temperature for 6 h. Subsequently, the mixture was heated at 90 °C for 2 h. The orange microcrystals were recovered by centrifugation, thoroughly washed with THF and dried under vacuum. Samples were kept in dark under argon atmosphere.

#### Synthesis of MnBr(dmbpy)(CO)_3_

The synthesis was adapted from reported procedures.^30^ In a round bottom flask, MnBr(CO)_5_ (40.7 mg, 0.148 mmol) and 5,5′-dimethyl-2,2′-bipyridine (30 mg, 0.163 mmol) were solubilized in diethyl ether (10 ml). The solution was refluxed at 40 °C for 3 h. The orange solid was recovered by filtration and washed with diethyl ether. Finally, it was dried at room temperature under vacuum.

#### Loading efficiency determination

The extent of metalation on UiO-67-bpy was determined *via* X-ray fluorescence (XRF), transmission electron microscopy-energy dispersive X-ray spectroscopy (TEM-EDX) and TGA.

#### CO detection

A suspension in THF of the sample to be analyzed was spin coated on a glass substrate. The weight of the sample was between 50 and 100 μg. The sample was placed in a custom-made chamber with a glass window on the top and irradiated with a 300 W xenon lamp (Asahi Spectra Max-303 equipped with a 300- to 600 nm ultraviolet-visible module and ×1.0 collimator lens). The light intensity was 0.38 mW cm^–2^. The released CO gas from **CORF-1** was carried to the CO detector (HALO 3 TM trace gas analyzer from Tiger Optics) by N_2_ flow (500 ml min^–1^ flow rate) (Fig. S11†).

#### Cell culture procedure

To prepare an extracellular stimulation scaffold, 1–1.5 mg of **CORF-1_small_79** crystals and PDMS layer were deposited on a 35 mm glass bottom dish (Skylight glass base dish, IWAKI) by spin coating. The 500 μM solution of COP-1 in DMSO was diluted by DPBS (+Ca, +Mg) to 1.0 μM. HeLa cells were cultured in the prepared glass bottom dish, including a PDMS/**CORF-1** layer, with 2 ml of cell culture medium (Dulbecco's Modified Eagle Medium supplemented with 10% fetal bovine serum). HeLa cells were incubated at 37 °C in a humidified atmosphere of 5% (v/v) CO_2_ in air for 1 day. Before stimulation, the cells were incubated with 1.5 ml of 1.0 μM COP-1 solution for 1 h to introduce COP-1 into the cells. Afterward, the COP-1 solution was replaced with DMEM and then incubated for 1 h at 37 °C.

#### Confocal fluorescence imaging

The illumination of the MOF-based substrate was carried out using white light (light power 150 W) for 90 seconds and then incubated for 10 min at 37 °C in 5% CO_2_ before imaging. All fluorescent images used for cell imaging were acquired on an Olympus FV-1000. The imaging parameters for the COP-1 were excitation at 488 nm and emission at 490–630 nm.

## Results and discussion

### Size controlled synthesis of **CORF-1** crystals

**Fig. 1:**
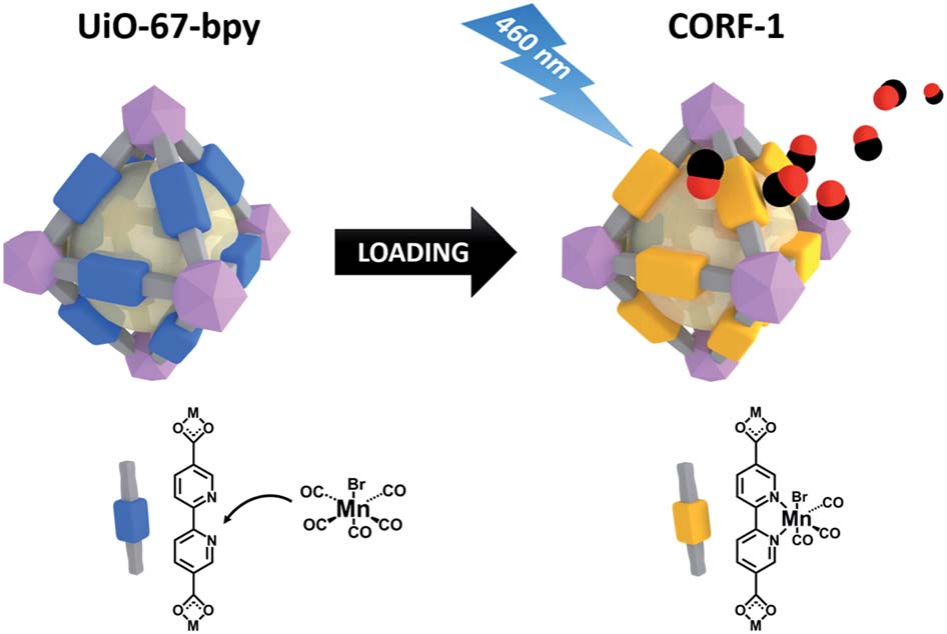
Schematic showing the loading of MnBr(bpy)(CO)_3_ on UiO-67-bpy to synthesize **CORF-1**, and the subsequent CO release upon light irradiation.

Inspired by previous reports on photoactive manganese carbonyl complexes, we sought to address the CO-releasing properties of the Mn(CO)_3_ moieties when coordinatively immobilized in a MOF structure. Our strategy to design the CO-releasing framework involves the zirconium-based UiO-67-type framework,^31^ Zr_6_O_4_(OH)_4_(bpydc)_6_, (UiO-67-bpy, bpydc = 5,5′-dicarboxylate-2,2′-bipyridine) as the starting material and the subsequent metalation of its open 2,2′-bipyridine (bpy) coordination centers to form the photoactive MnBr(bpy)(CO)_3_ core on the MOF walls, leading to MnBr(bpydc)(CO)_3_@UiO-67 (**CORF-1**: CORF = CO-releasing framework) (Fig. 1). The straightforward formation of **CORF-1** using MnBr(bpydc)(CO)_3_ as the metallo-ligand was not successful due to the decomposition of the manganese complex at elevated temperature (120 °C).^31^ Note that a similar Mn-containing framework based on UiO-67-bpy with the mixed composition (1/1) between bpydc and bpdc (biphenyl-4,4′-dicarboxylic acid) was previously reported;^32^ however, the mixed ligand framework limits the extent of metalation. Herein, we use only bpydc for the synthesis of **CORF-1** and try to achieve a very high CO loading capacity.

**Fig. 2:**
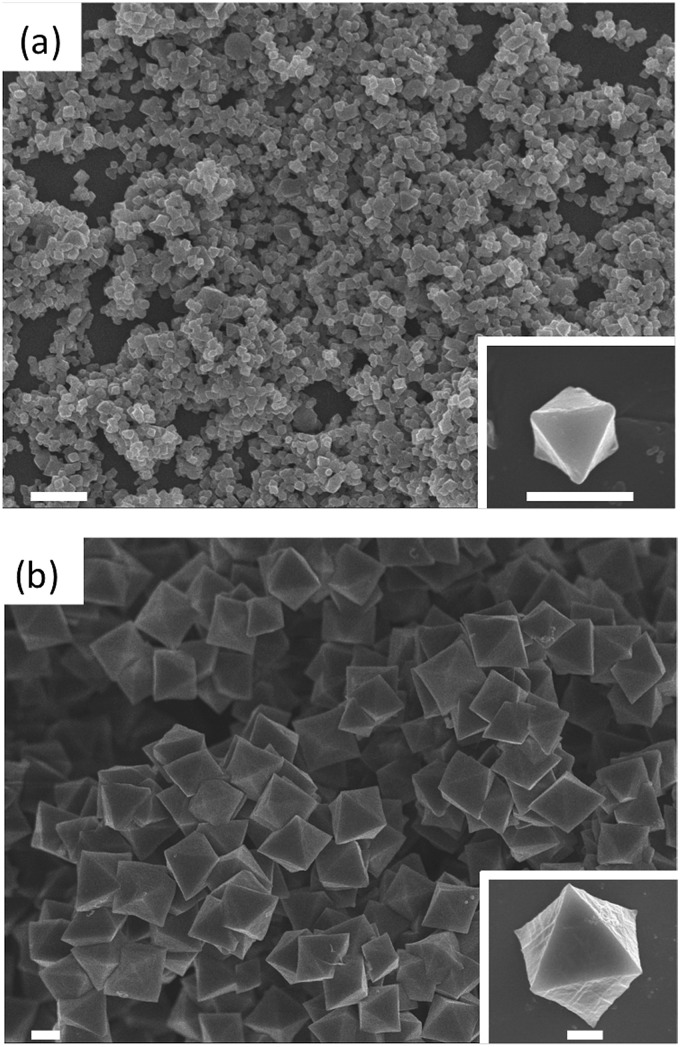
Representative FESEM of UiO-67-bpy synthesized with 30 eq. (a) and 90 eq. (b) of HAc. Scale bars: 1 μm and 500 nm (inset).

First, we optimized the synthesis conditions to afford highly crystalline UiO-67-bpy with tunable sizes (Fig. S1†). The bpydc ligand (20 mM) and ZrCl_4_ (20 mM) were reacted in DMF at 120 °C in the presence of 30 or 90 equivalents of acetic acid (HAc) as a modulator. As expected, the amount of HAc in the reaction had a significant impact on the crystal size of UiO-67-bpy; when 30 eq. of HAc were used, the crystals obtained had a mean size of 260 nm ± 80 nm, whereas the mean size of the crystals increased to 1200 nm ± 180 nm with increasing the amount of modulator to 90 eq. (Fig. 2).

After washing and activating the UiO-67-bpy crystals, the post-synthetic metalation of MnBr(CO)_5_ was carried out. Initially, the crystals were immersed in a THF/toluene (1 : 1) solution of MnBr(CO)_5_ and heated at 90 °C for 2 h as previously described.^33^ Even though this process afforded the metalation of bpydc, its yield was poor (40%) as determined by X-ray fluorescence (XRF) experiments and transmission electron microscopy energy dispersive X-ray spectroscopy (TEM-EDX) (Table S1†). In order to increase the loading amount, we implemented a two-step loading process: first, the precursor solution containing MnBr(CO)_5_ was allowed to diffuse into the crystals for 6 h at RT and, subsequently, the temperature was raised to 90 °C for 2 h to promote the coordination reaction with bpy inside the MOF. This strategy allowed significantly higher yields of loading (Table S1, Fig. S2†). The extent of loading was also largely affected by crystal size; the smaller crystals had higher loading efficiency due to the shorter diffusion path. Thus, the loading of the smaller crystals reached 79% (hereafter called **CORF-1_small_79**), while in the case of the bigger crystals the loading was 60% (**CORF-1_big_60**). Interestingly, we observed that by repeating this process a second time on **CORF-1_small_79**, almost fully loaded samples with 95% loading efficiency (**CORF-1_small_95**) were obtained.

**Fig. 3:**
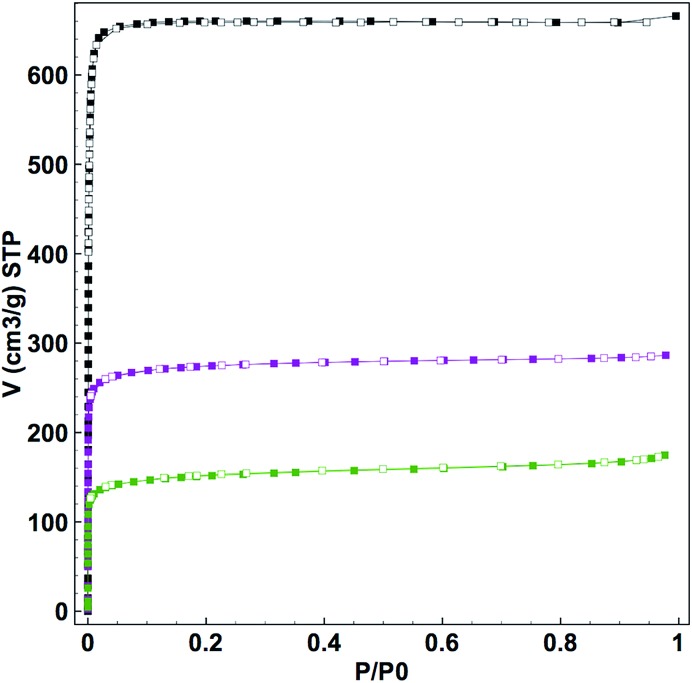
N_2_ adsorption isotherms performed at 77 K of UiO-67-bpy (black), **CORF-1_small_79** (purple), **CORF-1_small_95** (green). Filled and empty symbols correspond to adsorption and desorption respectively.

As confirmed by powder X-ray diffraction (PXRD) experiments and scanning electron microscope (SEM) observations, neither the crystallinity nor the morphology of the samples was altered by the loading process (Fig. S3 and S4†). In addition, the TEM-EDX analysis of all **CORF-1** samples revealed a homogeneous distribution of Mn(i) ions within the structure (Fig. S5†). The nitrogen adsorption experiment performed at 77 K indicated a decrease in BET surface area (BET) upon loading. The *S*_BET_ of the smaller crystals of UiO-67-bpy dropped from 2818 m^2^ g^–1^ to 1077 m^2^ g^–1^ and 586 m^2^ g^–1^ for **CORF-1_small_79** and **CORF-1_small_95**, respectively (Fig. 3). These results are consistent with the expected pore blocking caused by the MnBr(CO)_3_ incorporation within the UiO-67-bpy structure.

The formation of the MnBr(bpydc)(CO)_3_ moiety within **CORF-1** was evidenced *via* FTIR spectroscopy with the appearance of the characteristic CO stretching vibrations at 2032 and 1946 cm^–1^ (Fig. S6a†).^34^ In addition, the solid-state UV-Vis absorption spectrum revealed a new broad visible band (400–600 nm) ascribed to a metal-to-ligand charge transfer (MLCT) transition band, from the manganese centers to the bipyridine ligands (Fig. S6b†).

### Photoactive CO-release from **CORF-1**

**Fig. 4:**
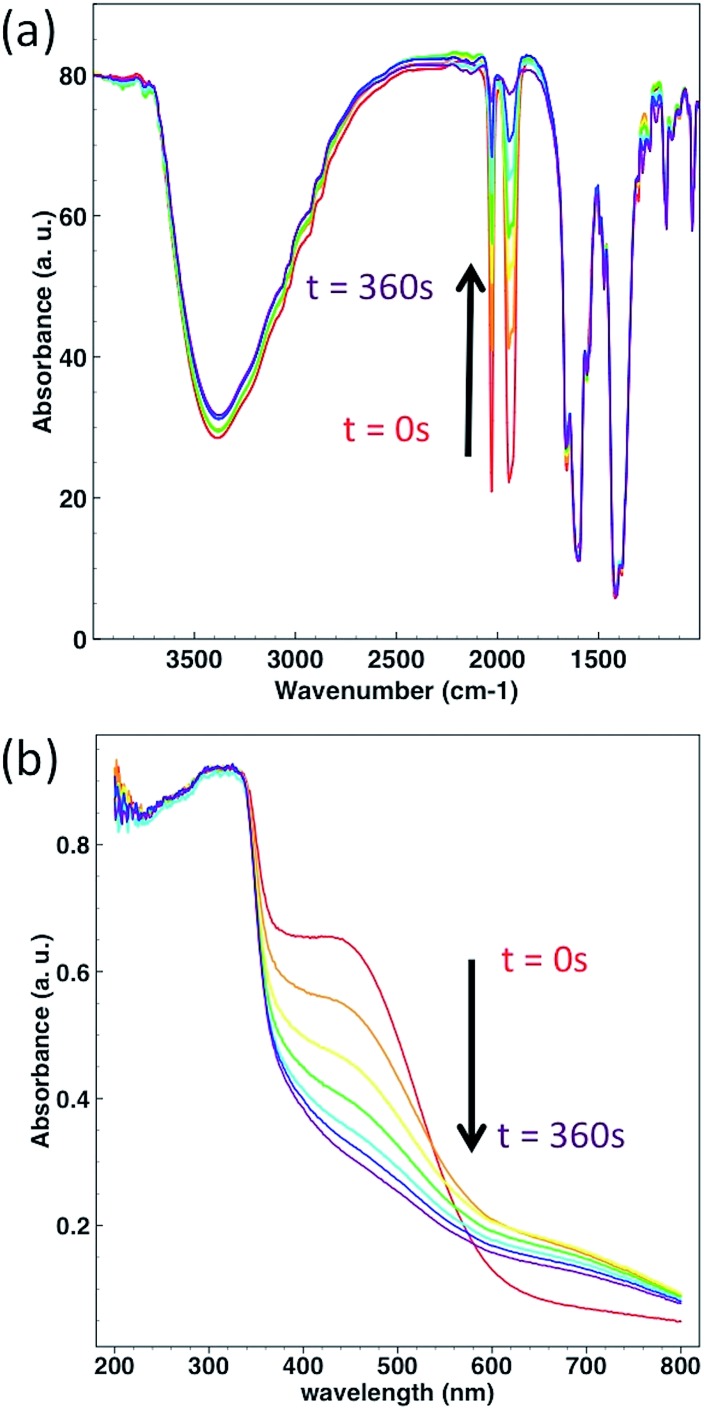
(a) Change in the FTIR spectra of **CORF-1_79** upon light irradiation. (b) Change in the solid state UV-Vis spectra of **CORF-1_79** upon light irradiation. Each spectrum was taken after 60 s of irradiation at 460 nm. Light power was 300 W.

In order to test the photoreactivity of **CORF-1** in the solid state, its response to visible light (460 nm and 300 W) was monitored *via* infrared and UV-Vis spectroscopies. Immediately after irradiation, the unambiguous disappearance of the characteristic CO stretching vibration started, indicating changes in the Mn(i) coordination sphere and becoming the first indicator of CO release (Fig. 4a). Along the same line, the MLCT absorption band (400–600 nm) progressively vanished under the same light exposure (Fig. 4b).

In contrast, when similar experiments were carried out with a prototypical molecular photoCORM of MnBr(dmbpy)(CO)_3_ (Fig. S7†: dmbpy = 5,5′-dimethyl-2,2′-bipyridine) in the solid-state, no change in the spectra was observed, revealing no sign of CO-release in the solid-state (Fig. S8†). This is remarkable, because Mn(i) has the same coordination environment in **CORF-1** as that in MnBr(dmbpy)(CO)_3_ and in both cases the MLCT band is centered at similar wavelengths. Note that the solution of MnBr(dmbpy)(CO)_3_Br efficiently released CO molecules when excited at 460 nm (Fig. S9†). Therefore, the difference in reactivity in the solid state must be attributed not to electronic structures but to the spatial arrangement of the photoactive species; the voids in the framework structure offer spatial segregation between the photoactive centers of the Mn(i) complex, preventing the aggregation-induced quenching observed in the molecular photoCORMs in the solid state. This is important, as one of the major limitations of photoCORMs for their practical application is the lack of solubility in aqueous media, which hinders their performance.^35^ The ability of **CORF-1** to release CO in the solid state avoids the need for solubilization and therefore paves the way for their use in colloidal form and their integration into extracellular devices.

**Fig. 5:**
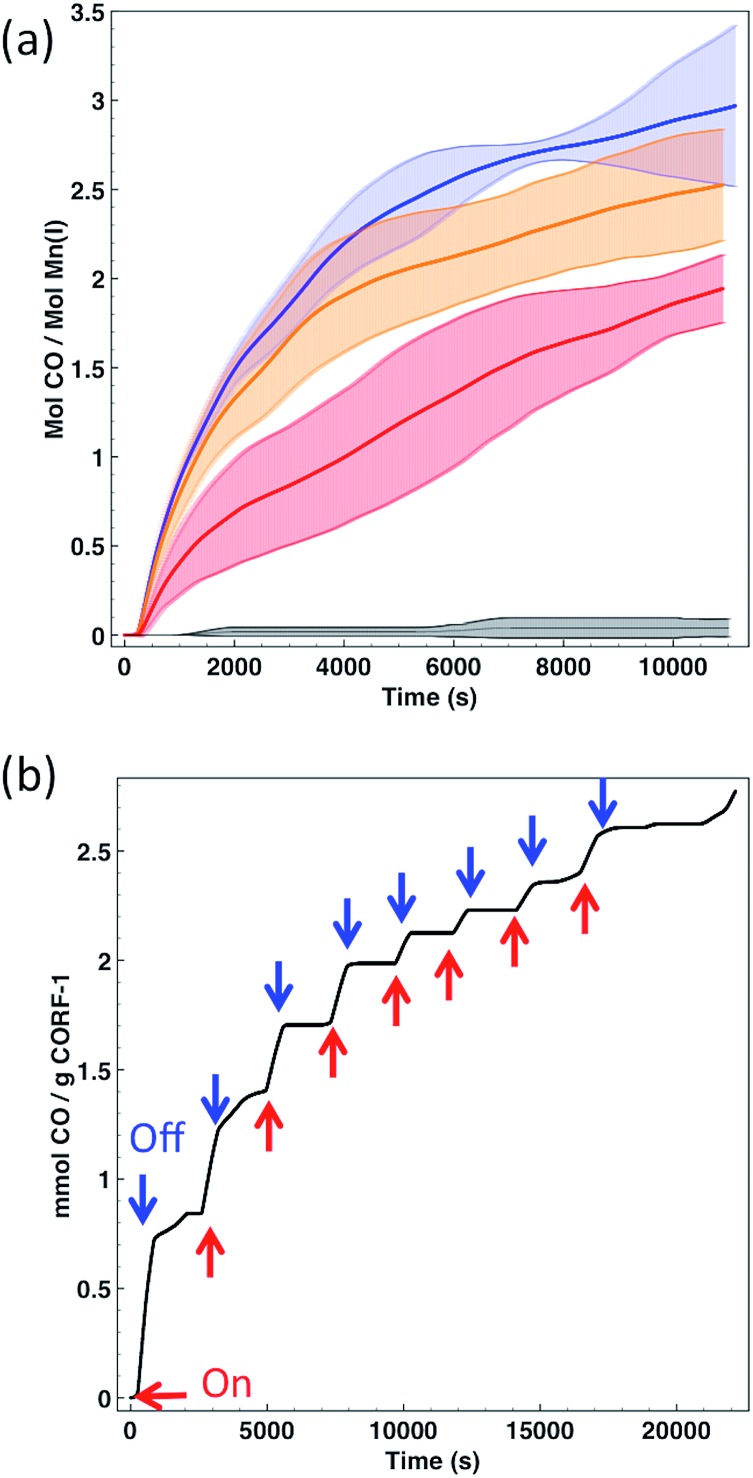
(a) Time-dependent release of CO to the gas phase per Mn(i) for **CORF-1_small_79** (blue), **CORF-1_small_95** (orange), **CORF-1_big_60** (red) and [Mn(dmbpy)(CO)_3_Br] (black). Thick lines represent the average value, while dashed intervals show the standard error of three independent replicates. (b) CO release profile of **CORF-1_small_79** upon intermittent irradiation at 460 nm and 15 W.

In order to quantify the CO release from the **CORF-1**, we attempted to use the conventional myoglobin assay, which is based on the UV-Vis spectroscopic detection of the conversion of deoxyMb to COMb (Mb = myoglobin) (Fig. S10†). Though we detected the efficient release of CO by this method, it was observed that the extent of leaching of the Mn(i) complex from the **CORF-1** into the solution was too high to unambiguously attribute the CO release solely to the **CORF-1** and not to the leached photoreactive by-products (almost 50% of leaching). This leaching is most likely attributed to the framework decomposition by phosphate buffer saline (PBS) that was incorporated into the buffer solution for myoglobin assay. In order to overcome this shortcoming, an in-line CO detector was customized to detect the CO released from **CORF-1** in the solid state (Fig. S11†). Thus, the spin-coated samples of **CORF-1** crystals were irradiated at 460 nm with 15 W of light power to promote the CO release. The liberated CO was transferred by N_2_ carrier gas to the in-line detector. The release efficiencies of CO from either **CORF-1_small_79**, **CORF-1_small_95**, **CORF-1_big_60** or the molecular complex [Mn(dmbpy)(CO)_3_Br] were tested and compared (Fig. 5a and S12†). **CORF-1_small_79** has an average release of 2.96 molecules of CO per Mn(i) ion and 4.65 mmol CO per g(**CORF-1**), which translates into an average photoreleasing efficiency of 99%. The average CO release of **CORF-1_small_95** and **CORF-1_big_60** was estimated to be 2.52 and 1.94 molecules of CO per Mn(i) ion, respectively, which translates into a photoreleasing efficiency of 84% and 65%, respectively. Note that these values are among the highest reported for photoCORMAs (Table S3†). On the other hand, no significant release from MnBr(dmbpy)(CO)_3_ was detected, in accordance with the results from infrared and UV-Vis spectroscopies described above.

In light of these results three main conclusions can be drawn: (i) in the solid-state, the MnBr(dmbpy)(CO)_3_ moiety only releases CO molecules upon light irradiation when it is anchored to the wall of the **CORF-1** structure; (ii) as evidenced by the performance of **CORF-1_small_79**, all three CO molecules coordinated to the Mn(i) ion are released, and (iii) light penetration is the limiting parameter in the photoefficiency of **CORF-1**. Assuming that there is a concentration gradient of photoactive centers from the surface to the core of the crystal when the loading is not fully achieved, the differences in the CO release between **CORF-1_small_79** and **CORF-1_small_95** can be explained by the fact that light is not able to reach the photoactive centers located in the core of the crystal. This effect is more evident in the case of larger crystals of **CORF-1_big_60** where only 65% of CO was released due to the lower surface to volume ratio in the bigger crystals. Once the photo-triggered release of CO from **CORF-1** was demonstrated and quantified, we aimed to demonstrate the temporal control of the release of CO. As shown in Fig. 5b, CO was only evolved from **CORF-1** with light irradiation and the release immediately stopped when light was switched off. Remarkably, the **CORF-1** structure was maintained in all cases after the releasing experiments, as evidenced by the XRPD experiments (Fig. S13†).

### Cell stimulation experiments based on **CORF-1** embedded cell culture substrate

**Fig. 6:**
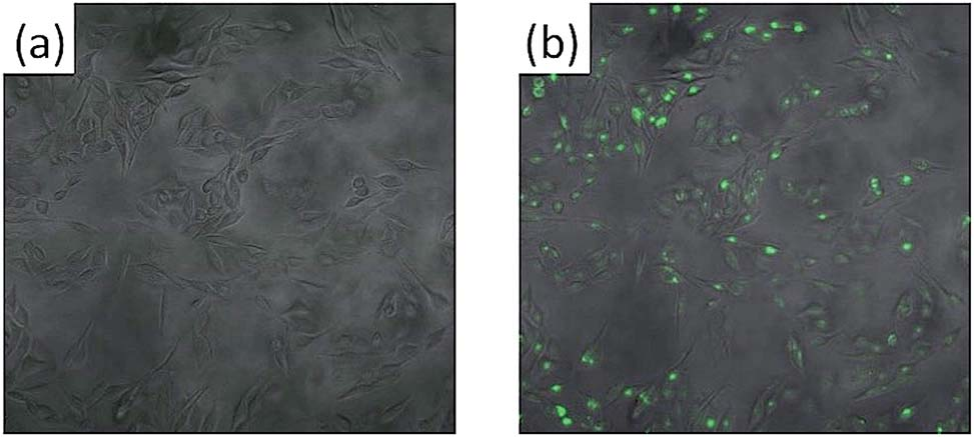
Merged confocal and transmission image of cultivated HeLa cells on the PDMS-embedded **CORF-1_small_79** before (a) and after (b) light irradiation at 460 nm. Scale bar: 100 μm. Green fluorescence coming from the COP-1 probe.

This high degree of control over CO release in the solid state prompted us to demonstrate the biological applicability of **CORF-1** as an extracellular scaffold. To this end, we prepared **CORF-1**-based substrates for cell cultures and microscopic imaging; a suspension of **CORF-1_small_79** crystals was spin-coated on a glass-bottomed culture dish. A second spin coating of a gas permeable and biocompatible polymer matrix (PDMS, polydimethylsiloxane) was embedded the **CORF-1_small_79** crystals. As we previously confirmed for the similar NO-release framework system,^24^ this configuration allowed the adhesion and culture of living cells on the top of the PDMS layer and gave no chance for embedded crystals to be dissolved into cell culture media, and thus promised almost no cytotoxicity. Before light irradiation experiments, the HeLa cells were treated with a turn-on fluorescent probe (COP-1) suitable for the intracellular detection of CO.^36^ The illumination of the MOF-based substrate by white light resulted in an unambiguous increase in the fluorescence of the HeLa cells treated with the COP-1 probe, as observed by confocal laser scanning microscopy (Fig. 6). Thus, we have demonstrated the intracellular delivery of CO upon visible light exposure, in which CO was released from **CORF-1** and was able to diffuse through PDMS and the culture media to reach the interior of the HeLa cells and react with the COP-1 probe.

## Conclusion

In summary, we have prepared new CO-releasing framework materials by the post-synthetic immobilization of photoactive manganese carbonyl centers onto the walls of a robust and porous zirconium-based MOF. Efficient and controllable light-induced CO release was demonstrated upon low intensity visible light exposure. Using the coordination modulation approach, the crystal size of **CORF-1** could be finely tuned from 260 nm up to 1 mm, which proved to be an additional way to control the extent of CO release by the crystals. Finally, we have evidenced the intracellular uptake of CO originating from a photoactive MOF. We believe that the proven capacity of **CORF-1** to release CO in the solid state, combined with the recent advances in the integration of MOFs on devices will pave the way for a next generation of smart CO-releasing materials. These new materials provide an easy-to-localize platform for the therapeutic release of CO directly to targeted tissues or organs, which could be employed in inflammation related diseases such as inflammatory bowel disease. The localized release of CO would provide the anti-inflammation effect, avoiding the toxic side effects of CO in the blood and lungs. Along this line, further efforts to trigger CO release at wavelengths with higher penetration depth (near infra-red), combined with an improved integration into biocompatible devices, are underway.
