# Supplementary material for: Light responsive metal–organic frameworks as controllable CO-releasing cell culture substrates
Source: Chem Sci. 2016 Dec 21;8(3):2381–6. doi: 10.1039/c6sc04824b (PMC5364997; doi:10.1039/c6sc04824b)
Supplement: Supplementary file 1 [file SC-008-C6SC04824B-s001.pdf]

Electronic Supplementary Material (ESI) for Chemical Science.
This journal is © The Royal Society of Chemistry 2016

Supplementary Information for

Light responsive metal-organic frameworks as a controllable CO-releasing cell culture substrate

Stéphane Diring,[a] *Arnau Carné-Sánchez,*[a] *JiChen Zhang,*[b] *Shuya Ikemura,*[b] *Chiwon Kim,*[a,b] *Hiroshi Inaba,*[a,b] *Susumu Kitagawa,*[a,b,]* *Shuhei Furukawa*[a,]*

[a]Institute for Integrated Cell-Material Sciences (WPI-iCeMS), Kyoto University, Yoshida, Sakyo-ku, Kyoto 606-8501, Japan.

[b]Department of Synthetic Chemistry and Biological Chemistry, Graduate School of Engineering, Kyoto University, Katsura, Nishikyo-ku, Kyoto 615-8510, Japan.

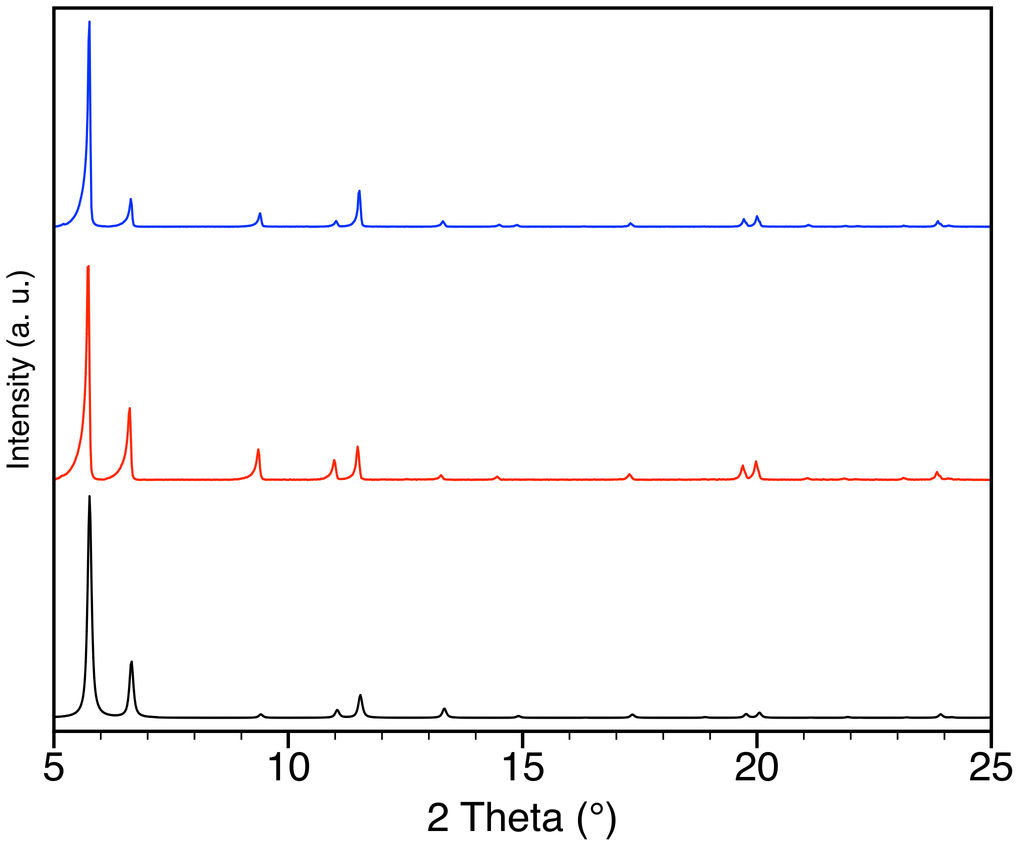

Figure S1. Simulated PXRD of UiO-67 (black) and experimental UiO-67-bpy synthesized in the presence of 30 eqv (red) and 90 eqv (blue) of acetic acid as modulators.

Table S1. Loading efficiency of the samples used in this study.

Sample	XRF	EDX
**CORF-1_Small_Non incubated***	39.6 ± 0.9	42.0 ± 4.5
**CORF-1_Small_79**	79.3 ± 0.5	77.3 ± 2.8
**CORF-1_Small_95**	95.1 ± 1.4	90.3 ± 7.0
**CORF-1_BIG_60**	60.1 ± 1.4	59.3 ± 6.1

*Sample prepared by directly heating at 90°C for 2 h a mixture of $MnBr(CO)_5$ and UiO-67. Error are obtained from the standard deviation of three replicates.

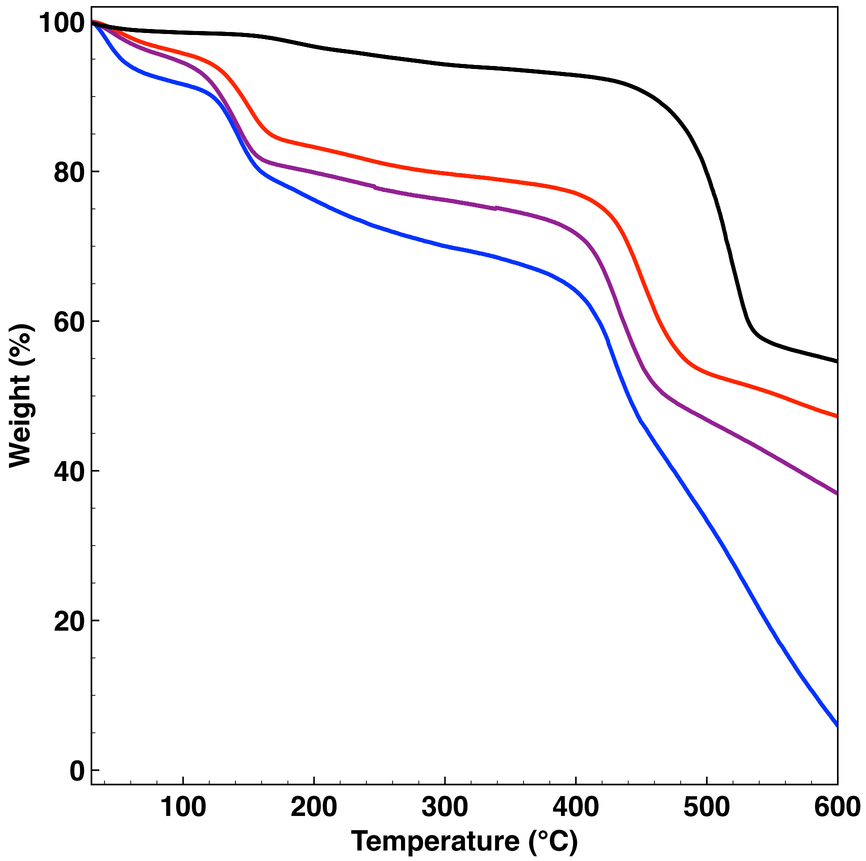

Figure S2. TGA analysis of UiO-67-bpy (black), **CORF-1_small_79** (blue), **CORF-1_small_95** (purple) and **CORF-1_big_60** (red). All CORF-1 samples show a two step decomposition process. The first one, between 80°C – 160°C, corresponds to the loss of CO molecules.[1] Decomposition of the framework starts at 400°C.

Table S2. Expected and experimental weight loss between 80 °C – 160 °C, attributed to the thermally induced release of CO, for the samples used in this study.

Sample	Theoretical weight loss (%)	Experimental weight loss (%)
**CORF-1_Small_79**	12.62	12.86
**CORF-1_Small_95**	14.11	13.86
**CORF-1_BIG_60**	10.31	11.37

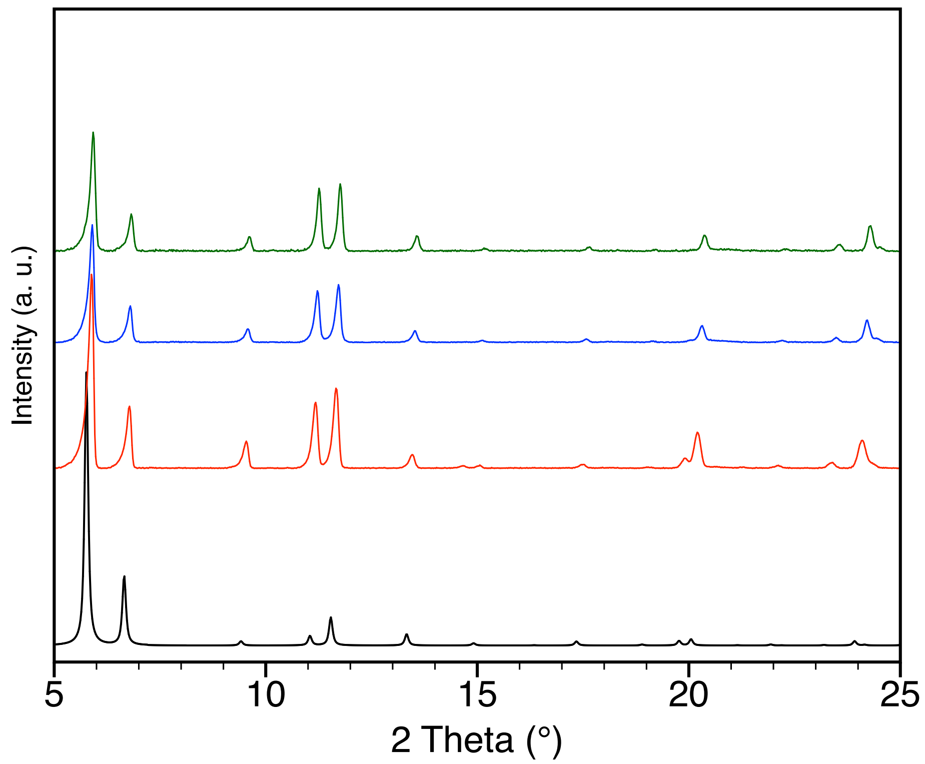

Figure S3. Simulated PXRD of UiO-67 (black) and experimental **CORF-1_big_60** (red), **CORF-1_small_79** (blue), **CORF-1_small_95** (green).

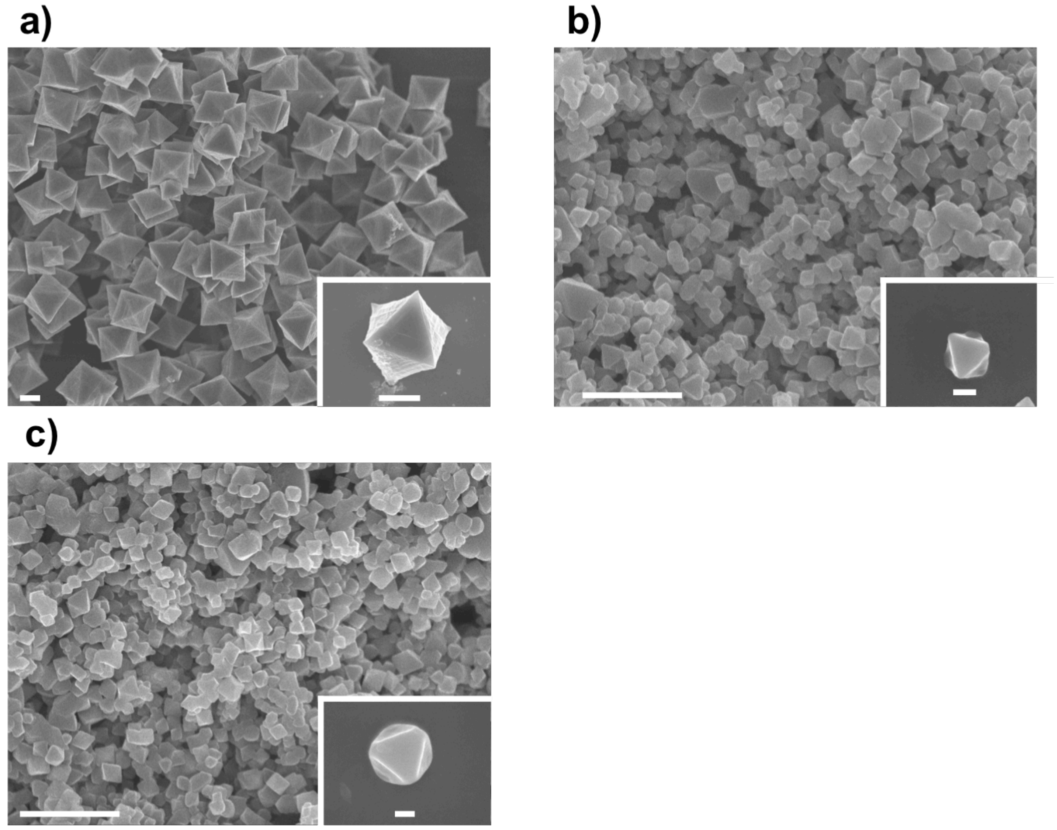

Figure S4. Representative FESEM images of **CORF-1_big_60** (a), **CORF-1_small_79** (b), **CORF-1_small_95** (c). Scale bar 1 μm and 500 nm (inset a) and 100 nm (inset b and c).

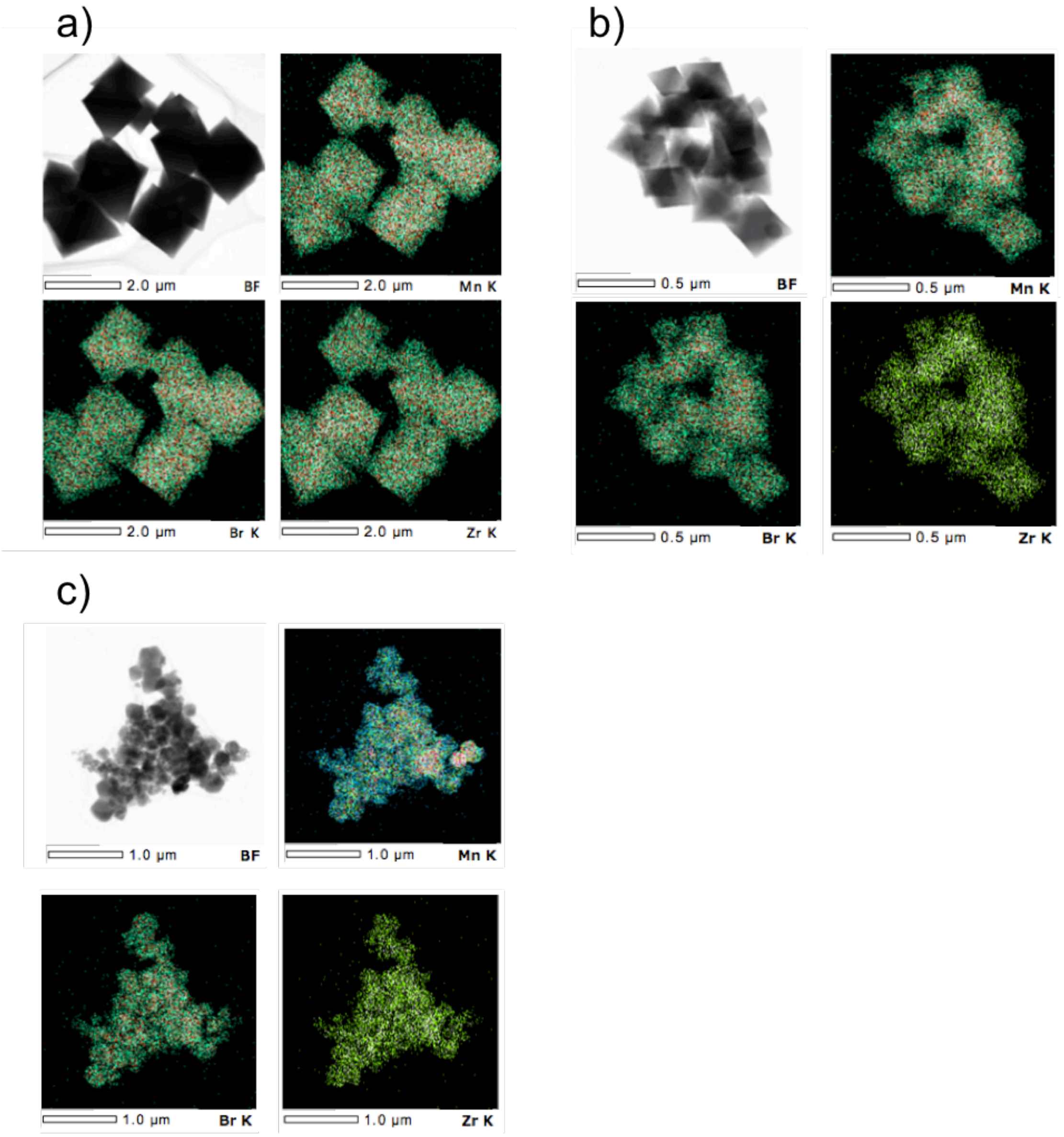

Figure S5. Representative TEM-EDX mapping of **CORF-1_big_60** (a), **CORF-1_small_79** (b), **CORF-1_small_95** (c).

a) b)

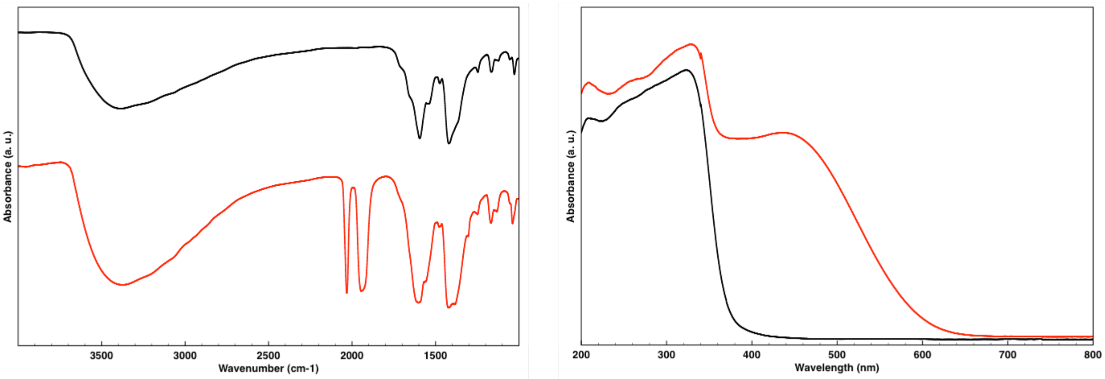

Figure S6. a) FTIR of UiO-67-bpy (black) and **CORF-1** (red). b) Solid state UV-VIS spectra of UiO-67-bpy (black) and **CORF-1** (red).

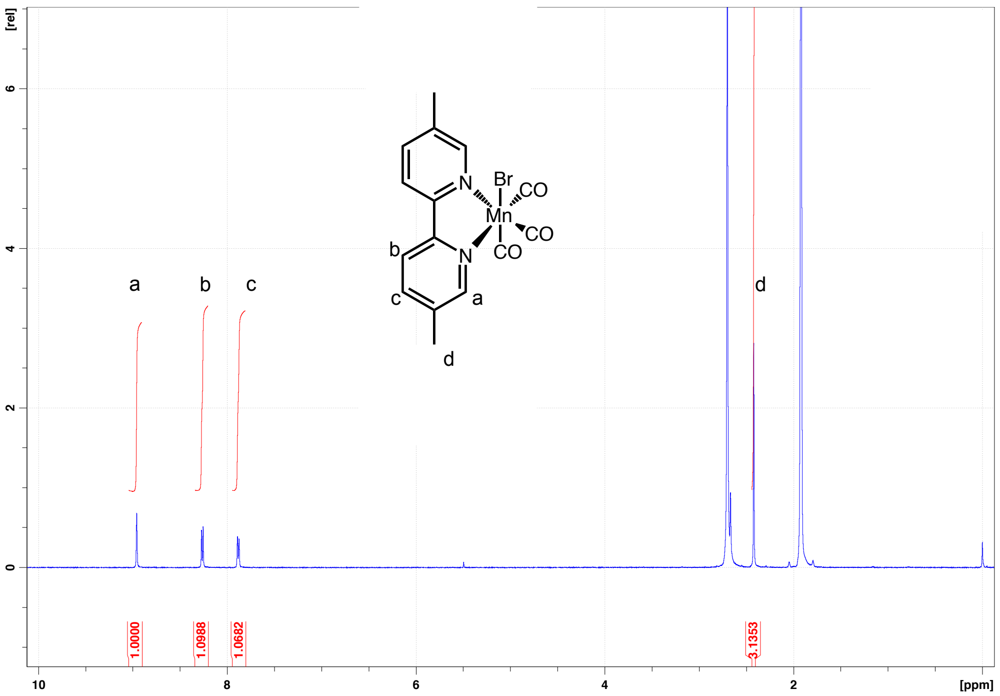

Figure S7. NMR of MnBr(dmbpy)$(CO)_3$ in acetone-d^6.

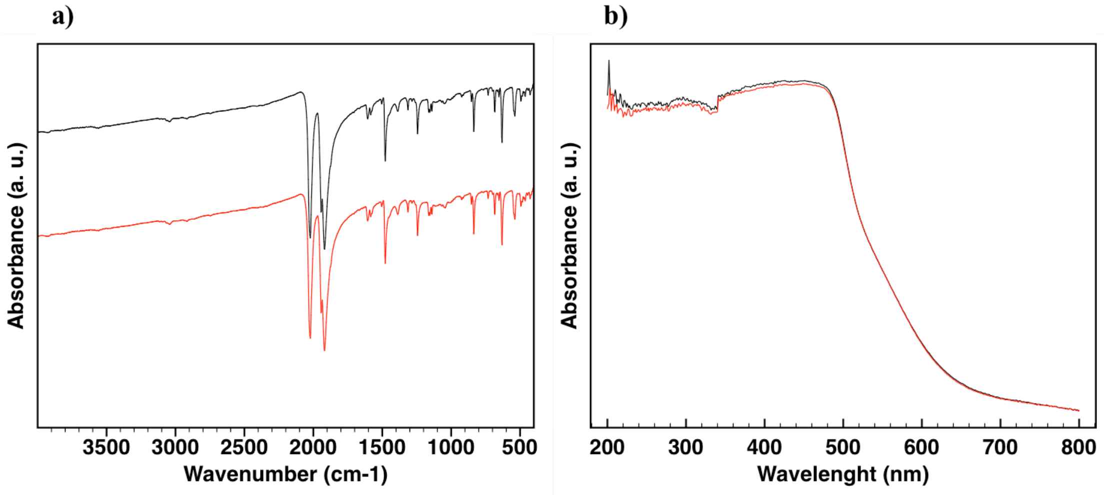

Figure S8. (a) FTIR spectra of $MnBr(dmbpy)(CO)_3$ before (black) and after (red) irradiation at 460 nm for XX min. (b) UV-VIS spectra of $MnBr(dmbpy)(CO)_3$ before (black) and after (red) irradiation at 460 nm for 90 min at 300 w of light power.

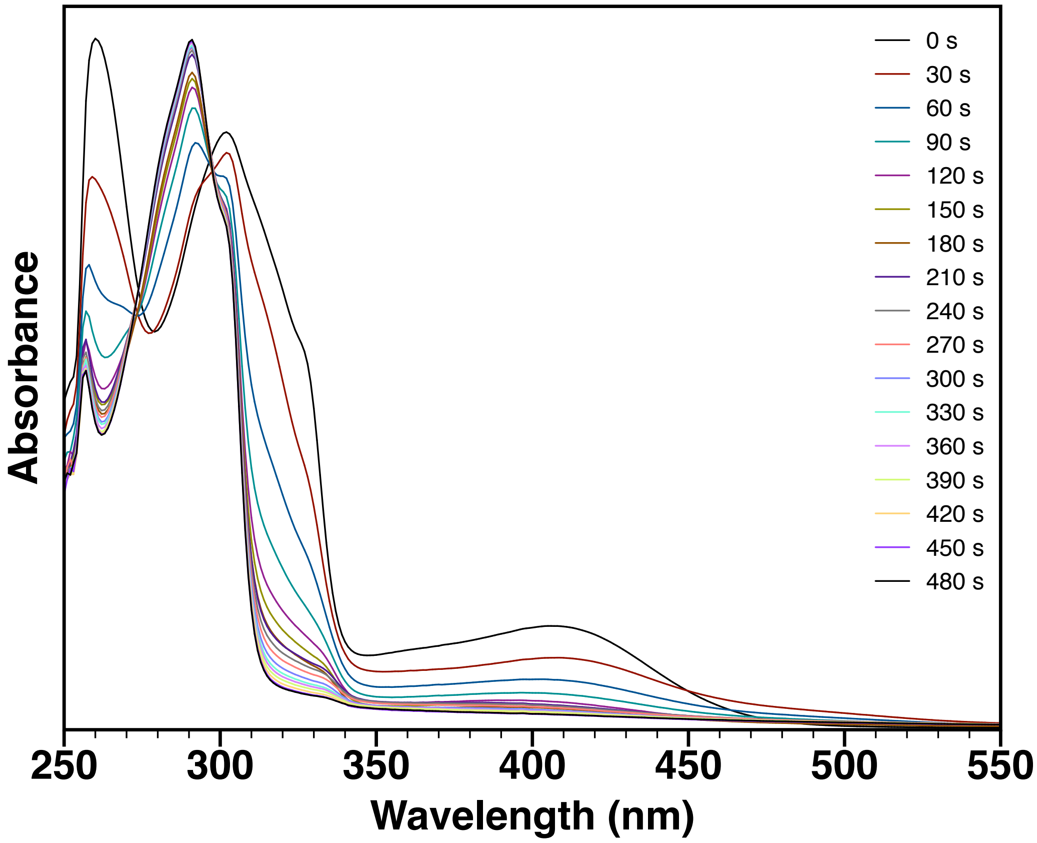

Figure S9. Changes in the UV-Vis spectra of a solution of MnBr(dmbpy)$(CO)_3$ (0.04 mM) in DMSO solvent upon irradiation at 460 nm. Legend indicates the irradiation time.

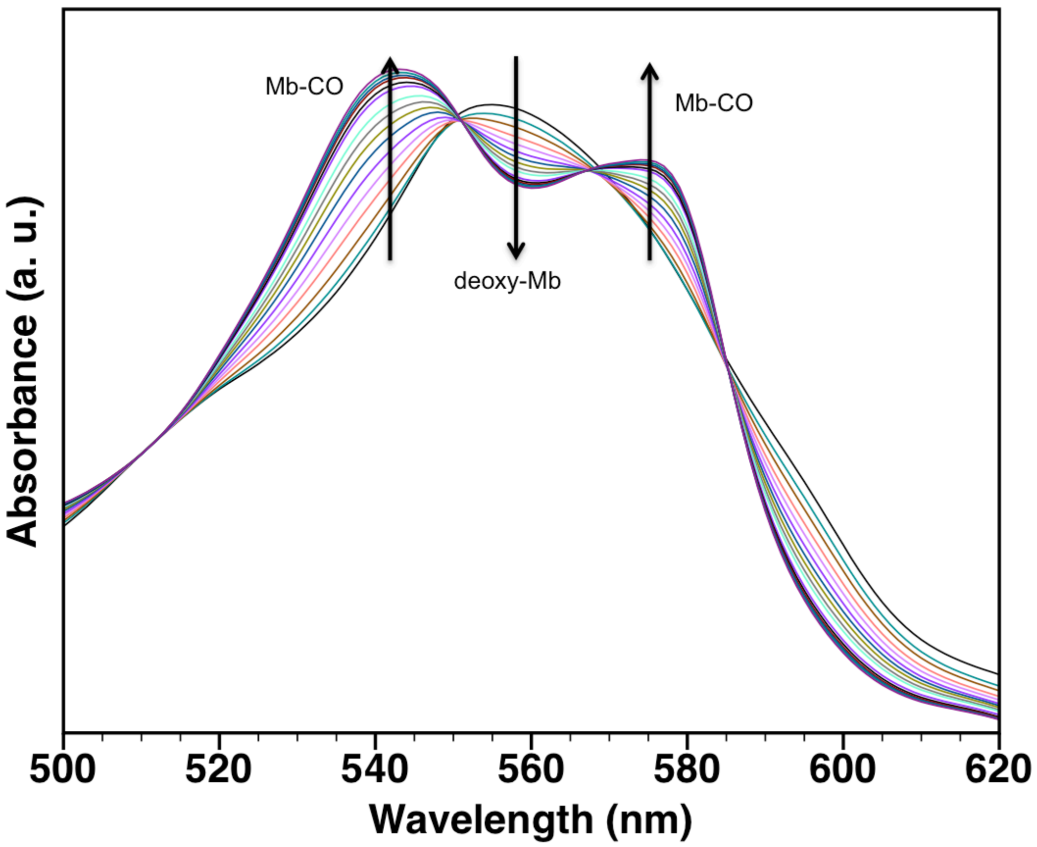

Figure S10. Myoglobin essay performed on **CORF-1_big_60**. Change of the adsorption of Q-band region of myoglobin with increased irradiation time at 460 nm for a suspension containing 100 μg of **CORF-1_big_60** in PBS buffer (pH = 7.4) in the presence of myoglobin (60 μM). Each spectra is taken after 30 s of irradiation

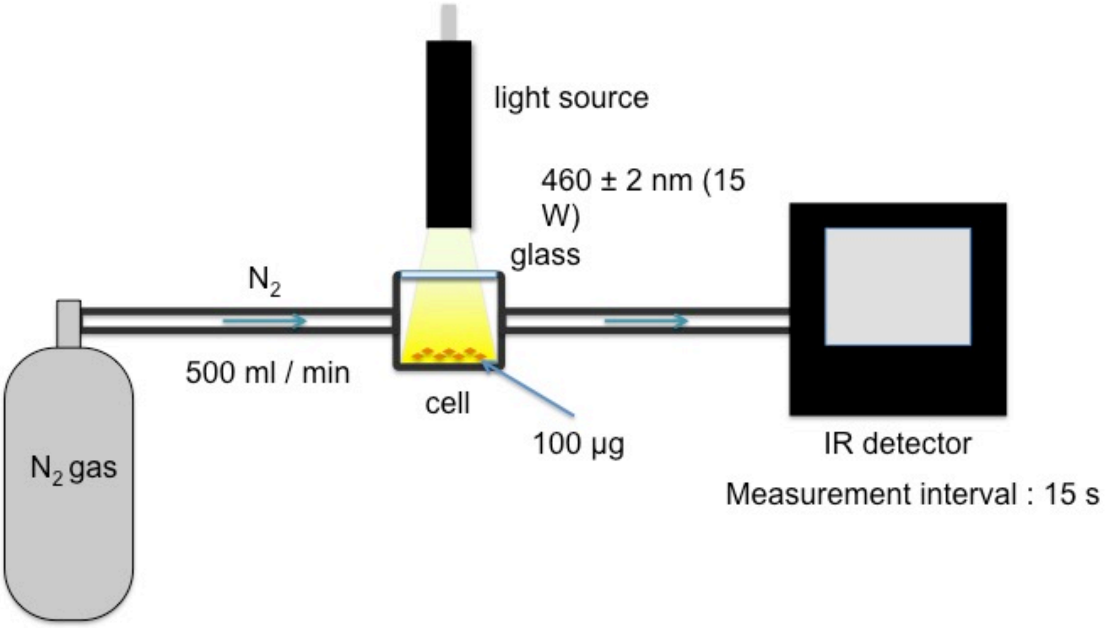

Figure S11. Customized in line CO detection system.

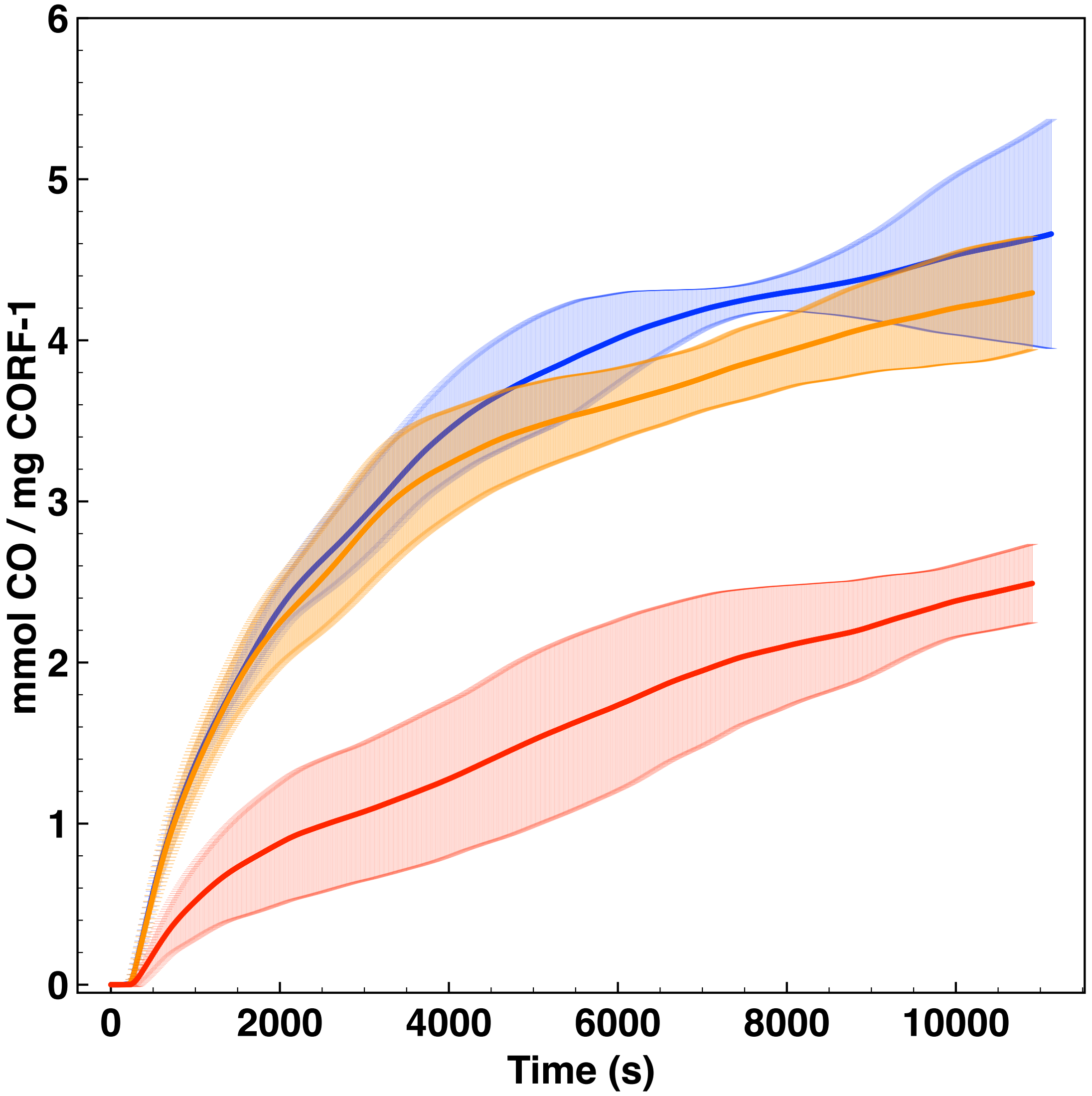

Figure S12. Time dependent amount of CO released to the gas phase per gram of **CORF-1_big_60** (red), **CORF-1_small_79** (blue), **CORF-1_small_95** (orange)**.**

Table S3. Performance of relevant photoCORMAs

Scaffold	CO loading (mmol/g)	λ of CO release (nm)	CO release (mmol/g)	ref
**Nanodimond**	0.1	365	-	2
**SiO_2 nanoparticle**	0.273	365	0.18-0.27	3
**Metallodendrimer**	12.47-17.84	410	7.85 – 11.76	4
**Protein cage**	-	456	-	5
**Mesoporous silica**	1.13	Broad visible lght	-	6
**Polymeric fibers**	26.94	365	3.1-3.7	7
**CORF-1_Small_79**	4.70		4.66	This work

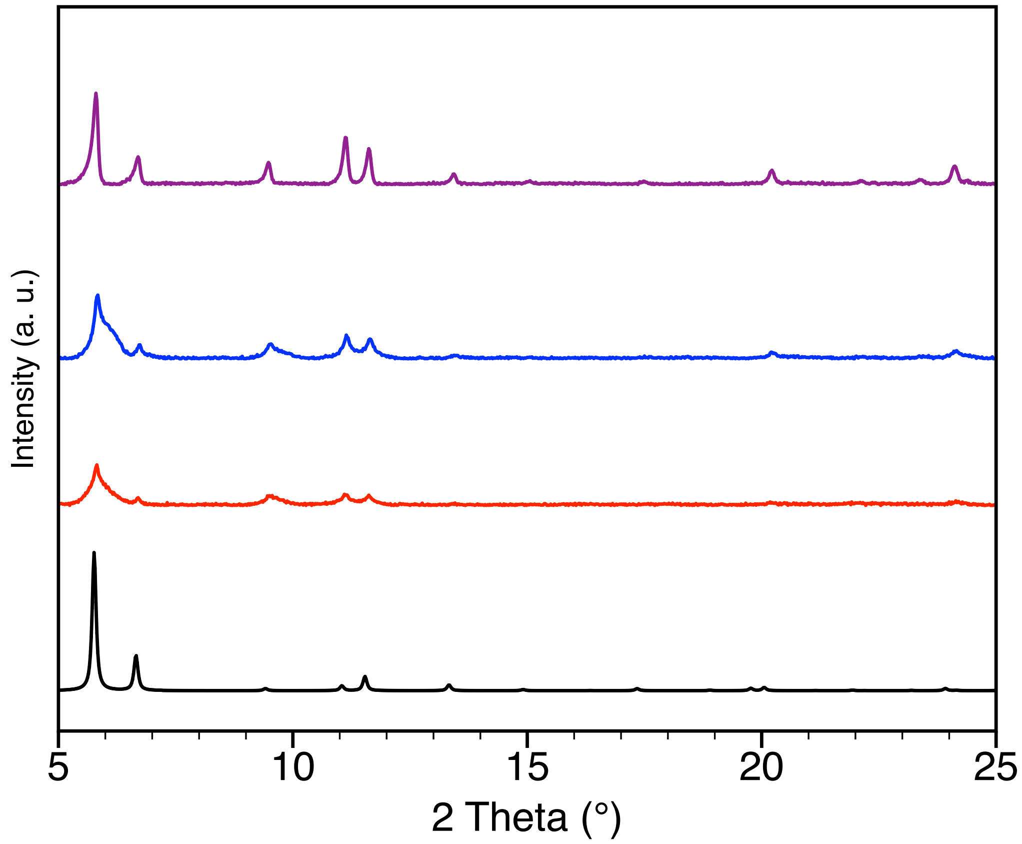

Figure S13. Simulated PXRD of UiO-67 and experimental **CORF-1_big_60** (purple), **CORF-1_small_79** (blue), **CORF-1_small_95** (red) after photo releasing experiments.

References.

S1. H. Fei, M. D. Sampson, Y. Lee, C. P. Kubiak, S. M. Cohen, *Inorg. Chem.* 2015, **14**, 6821

S2. G. Dordelmann, T. Meinhardt, T. Sowik, A. Krueger, U. Schatzschneider, *Chem. Commun.* 2012, **48**, 11528.

S3. G. Dördelmann, H. Pfeiffer, A. Birkner, U. Schatzschneider, *Inorg. Chem.* 2011, 50, 4362.

S4. P. Govender, S. Pai, U. Schatzschneider, G. S. Smith, *Inorg. Chem.* 2013, **52**, 5470.

S5. K. Fujita, Y. Tanaka, S. Abe, T. Ueno, *Angew. Chem. Int. Ed.* 2016, **55**, 1056. S6.

S6. M. A. Gonzales, H. Han, A. Moyes, A. Radinos, A. J. Hobbs, N. Coombs, S. R. J. Oliver, P. K. Mascharak, *J. Mater. Chem B.* 2014, **2**, 2107

S7. C. Bohlender, S. Glaser, M. Klein, J. Weisser, S. Thein, U. Neugebauer, J. Popp, R. Wyrwa, A. Schiller, *J. Mater. Chem.* B 2014, **2**, 1454.

Full author list for reference 11

11. A. R. Marques, L. Kromer, D. J. Gallo, N. Penacho, S. S. Rodrigues, J. D. Seixas, G. J. L. Bernardes, P. M. Reis, S. L. Otterbein, R. A. Ruggieri, A. S. G. Gonçalves, A. M. L. Gonçalves, M. N. D. Matos, I. Bento, L. E. Otterbein, W. A. Blättler, C. C. Romão, *Organometallics* 2012, **31**, 5810.
